# Expression of VEGF_*xxx*_b, the inhibitory isoforms of VEGF, in malignant melanoma

**DOI:** 10.1038/sj.bjc.6603839

**Published:** 2007-06-26

**Authors:** R O Pritchard-Jones, D B A Dunn, Y Qiu, A H R Varey, A Orlando, H Rigby, S J Harper, D O Bates

**Affiliations:** 1 Microvascular Research Laboratories, Department of Physiology, Preclinical Veterinary School, University of Bristol, Bristol, UK; 2 Department of Plastic Surgery, Frenchay Hospital, Bristol, UK; 3 Department of Pathology, Frenchay Hospital, Bristol, UK

**Keywords:** melanoma, VEGF_165_b, VEGF_*xxx*_b, metastasis

## Abstract

Malignant melanoma is the most lethal of the skin cancers and the UK incidence is rising faster than that of any other cancer. Angiogenesis – the growth of new vessels from preexisting vasculature – is an absolute requirement for tumour survival and progression beyond a few hundred microns in diameter. We previously described a class of anti-angiogenic isoforms of VEGF, VEGF_*xxx*_b, that inhibit tumour growth in animal models, and are downregulated in some cancers, but have not been investigated in melanoma. To determine whether VEGF_*xxx*_b expression was altered in melanoma, PCR and immunohistochemistry of archived human tumour samples were used. In normal epidermis and in a proportion of melanoma samples, VEGF_*xxx*_b staining was seen. Some melanomas had much weaker staining. Subsequent examination revealed that expression was significantly reduced in primary melanoma samples (both horizontal and vertical growth phases) from patients who subsequently developed tumour metastasis compared with those who did not (analysis of variance (ANOVA) *P*<0.001 metastatic *vs* nonmetastatic), irrespective of tumour thickness, while the surrounding epidermis showed no difference in expression. Staining for total VEGF expression showed staining in metastatic and nonmetastatic melanomas, and normal epidermis. An absence of VEGF_*xxx*_b expression appears to predict metastatic spread in patients with primary melanoma. These results suggest that there is a switch in splicing as part of the metastatic process, from anti-angiogenic to pro-angiogenic VEGF isoforms. This may form part of a wider metastatic splicing phenotype.

Predicting tumour metastasis is one of the main goals of the examination of the primary tumour. Most patients presenting with melanoma (85%) are without clinically detectable metastasis at primary excision. For these patients, histological examination is vital for staging and obtaining a relative risk of future metastasis. Both level of invasion within the skin (Clark's staging; Clark *et al*, 1969) and, more accurately, thickness of the primary lesion (Breslow thickness; Breslow, 1970; Balch *et al*, 2001b) correlate to disease progression and survival. These prognostic indicators of thickness and invasion are the most commonly used and indeed the basis for staging melanoma and the development of management strategies in terms of excision margin, sentinel node biopsy, further investigation and follow-up. The other key indicator of prognosis is the presence of ulceration. Patients with tumour thickness >4 mm have a 10-year survival of between 54 and 32% (Stadelmann *et al*, 1998; Balch *et al*, 2001a; Cook *et al*, 2002). Prognostic indicators to detect the patients, and especially the 13% of patients with thin melanoma (<1 mm), who will go on to develop metastasis are currently being investigated, and much work has been focused on the density of lymphatics surrounding primary malignant melanomas (Dadras *et al*, 2003; Shields *et al*, 2004).

A number of studies have attempted to investigate angiogenic growth factors to determine whether they can predict metastasis, but there is poor correlation between the most potent angiogenic factor vascular endothelial growth factor-A (VEGF-A) and metastatic spread (Turley *et al*, 1998; Straume and Akslen, 2001; Simonetti *et al*, 2002; Giatromanolaki *et al*, 2003; Potti *et al*, 2003, 2004; Stefanou *et al*, 2004; Yang *et al*, 2004). Expression of VEGF-A is an attractive potential marker of prognosis owing to its consistent upregulation in cancer (Carmeliet, 2000), including melanoma (Bayko *et al*, 1998; Bayer-Garner *et al*, 1999), but there are conflicting data surrounding the prognostic implication of any observed upregulation. Vascular endothelial growth factor-A exists in most tissues, including cancer, as multiple isoforms generated by alternative splicing of a single gene product. These isoforms are termed according to the amino-acid number (VEGF_121_, VEGF_143_, VEGF_165_, VEGF_189_ and VEGF_206_; Ferrara, 1999). The predominant molecular species produced by a variety of normal physiological and pathological tissues is VEGF_165_.

In 2002, we described the first isoform of a novel family of splice variants formed by the use of a distal splice acceptor site in the terminal exon of the VEGF-A gene encoding an open reading frame of identical length to the classical, proximally spliced isoforms, and resulting in proteins of the same length, but with an alternate C terminus. The mRNA encoding this isoform, termed VEGF_165_b (Bates *et al*, 2002; Figure 1), was demonstrated in renal cortical tissue, and almost all other normal tissues examined. Subsequent work has identified other isoforms using this distal splice site (DSS), including VEGF_121_b, VEGF_189_b and VEGF_145_b in normal human tissues (Woolard *et al*, 2004; Perrin *et al*, 2005). This alternative family of isoforms is termed the VEGF_*xxx*_b family, in contrast with the VEGF_*xxx*_ family where *xxx* denotes the amino-acid number (Perrin *et al*, 2005). Expression of VEGF_165_b mRNA was downregulated in renal cell carcinoma (Bates *et al*, 2002), prostate cancer (Woolard *et al*, 2004) and the vitreous of patients with diabetic retinopathy (Perrin *et al*, 2005), all angiogenic conditions. Subsequently, VEGF_165_b protein was shown to significantly and dose dependently inhibit VEGF_165_-mediated proliferation and migration of endothelial cells, vasodilatation of mesenteric arteries and angiogenesis in two different models of VEGF_165_-driven blood vessel growth (Woolard *et al*, 2004). Furthermore, it has been shown to inhibit hypoxia-driven angiogenesis in the retina (Konopatskaya *et al*, 2006). This family of isoforms has now been termed the anti-angiogenic family of VEGF isoforms, in contrast with the pro-angiogenic VEGF_*xxx*_ isoforms.

As the amino-acid structure of the VEGF_*xxx*_b family is 95–96% identical to VEGF_*xxx*_, all previous studies on melanoma have not distinguished between the pro- and anti-angiogenic isoforms. It is therefore possible that changes in VEGF expression in melanoma are due to changes in isoform expression, and it is this that predicts progression or metastasis. We therefore determined whether VEGF isoform expression was altered in melanoma.

## METHODS

Primary metastatic melanoma was defined as primary tumours removed from a patient who subsequently went on to develop metastatic disease, confirmed to be melanoma in origin. Nonmetastatic melanoma was defined as primary tumours removed from a patient who subsequently had not developed metastatic disease after at least 8 years. Samples were collected, and experiments carried out under Local Ethical Committee approval (North Bristol NHS Trust).

### Reverse transcription–PCR

Eight 5 *μ*m thick sections were cut from nine archival, formalin-fixed, paraffin-embedded primary melanoma specimens (0.88–8.1 mm thick) from Frenchay Hospital, Bristol. These were melted, rehydrated through alcohols and subjected to RNA extraction (Trizol extraction method) before reverse transcription to cDNA as previously described. The complementary DNAs, VEGF_165_ and VEGF_165_b, were then amplified using primers specific to these two isoforms (exon 7/8a for VEGF_165_: exon 7: 5′-GTAAGCTTGTACAAGATCCGCAGACG-3′, exon 8a: 5′-TCACCGCCTCGGCTTGTCACAT-3′; or exon 7/8b for VEGF_165_b exon 8b: 5′-TTAAGCTTTCAGTCTTTCCTGGTGAGACTGCA-3′), or primers that detect both isoforms – 3′ UTR: 5′-ATGGATCCGTATCAGTCTTTCCTGG-3′). For exon 7/8b primers, PCR cycles were 94°C for 30 s, 65°C for 30 s and 72°C for 30 s. For other primer pairs, annealing was carried out at 55°C. All PCR were carried out in 1.5 mM MgCl_2_, 25 *μ*M each of dCTP, dATP, dGTP and dTTP, 1 *μ*M of each primer, using *Taq* polymerase (Abgene, Epsom, UK), and 1 *μ*l of cDNA in 20 *μ*l volume in a Hybaid DNA Express PCR machine. Products were separated by agarose gel (3% in TAE) electrophoresis and visualised by ethidium bromide staining (0.5 *μ*g ml^−1^).

#### Surface plasmon resonance

To confirm the binding affinity of VEGF_165_ and VEGF_165_b to the VEGF_165_b-specific antibody, we amine coupled the latter to a CM5 sensor chip (Biacore AB, Uppsala, Sweden) to an immobilisation level of 630 response units (RU). Coupling was performed using EDC/normal horse serum (NHS) and 1 M ethanolamine (Biacore AB) as per the manufacturer's instructions, with the antibody dissolved in 10 mM sodium acetate (pH 4.5). A blank reference cell was formed by the same activation and deactivation process involved in amine coupling, but without adding any protein to be coupled to the surface. Samples, containing VEGF_165_ or VEGF_165_b diluted in HBS-EP sample buffer (HEPES-buffered saline with EDTA and P20 surfactant; Biacore AB) were then run at two-fold serial dilutions from 180 nM down, in random order, with duplicate concentrations. Injection was performed at 30 *μ*l min^−1^ for 3 min, followed by 6 min of buffer only. Regeneration was performed by injection of 4 M MgCl_2_ at 20 *μ*l min^−1^ for 40 s, followed by a 2-min period of stabilisation.

### Immunohistochemistry

#### Pan-VEGF immunohistochemistry

Sections were cut from 19 melanomas (9 metastatic and 10 nonmetastatic). These melanomas were matched for thickness and time since taken, as closely as possible. There was no significant difference between the thicknesses (nonmetastatic 2.8±1.2 mm, metastatic 4.0±0.9 mm, *P*>0.25, *t*-test). All tumour samples were archived tissues taken 5–12 years ago and stored as paraffin-embedded blocks. There was no statistical difference between the age of patients (mean±s.e.m. age 58.3±6.9 years (metastatic) *vs* 60.9±6.2 years (nonmetastatic) Mann–Whitney *t*-test *P*=0.7) and tumour ulceration (mean±s.e.m. incidence of ulceration 0.5±0.17 (metastatic) *vs* 0.33±0.16 (nonmetastatic) Mann–Whitney *t*-test *P*=0.2). The mean±s.e.m. follow-up time (time between excision of the tumour and the analysis of expression) was not significantly different between the metastatic melanoma and nonmetastatic melanoma (7.6±1.3 years, metastatic; 8.5±0.17 years, nonmetastatic; *P*>0.50 *t*-test).

A commercially available anti-VEGF antibody A-20 (rabbit anti-human VEGF antibody, Santa Cruz, CA, USA) was used to investigate expression of all isoforms of VEGF (including VEGF_*xxx*_b) – termed pan-VEGF. This antibody has previously been shown to detect both VEGF_*xxx*_b and VEGF_*xxx*_ isoforms, and both heparin-binding and nonheparin-binding isoforms (Woolard *et al*, 2004; Perrin *et al*, 2005). Serial sections (6 *μ*m) were cut from tissue blocks of excised melanoma from the melanoma archive at Frenchay Hospital, Bristol. Sections were dewaxed with xylene for 15 min before rehydration in graded alcohols. Antigen retrieval was performed with microwave treatment for 3 min at 800 W and 10 min at 120 W in 0.01 M sodium citrate buffer, pH 6.0, and the container closure adjusted to maintain the solution just below boiling point (96±2°C). Sections were immersed in 3% hydrogen peroxide for 5 min to block endogenous peroxidase. Then they were incubated with 1.5% NHS for 30 min in a humid chamber at room temperature to block nonspecific binding. After washing, each slide was subject to incubation with a pan-VEGF (A-20) rabbit antibody (Santa Cruz) at 5 *μ*g ml^−1^ NHS. Control IgG was used at 5 *μ*g ml^−1^ NHS. Sections were incubated overnight. The blocking step with NHS was repeated before the addition of the biotinylated horse anti-rabbit secondary antibody (2 *μ*g ml^−1^) for 30 min. Then avidin-biotinylated enzyme complex was prepared according to the manufacturer's instructions (Vector Laboratories, Burlingame, CA, USA) and incubated with sections for 30 min at room temperature. This was followed by the addition of the visualising DAB substrate for all sections and counterstaining in haematoxylin. Sections were dehydrated in graded alcohols (70, 90 and 100% ethanol) before final clearing in xylene and mounting with DPX and glass coverslips. Slides were dried overnight before light microscopy examination.

### VEGF_*xxx*_b immunohistochemistry

**Figure 2:**
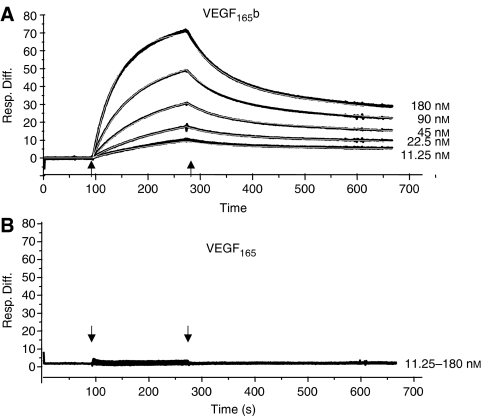
Biacore analysis of recombinant human VEGF_165_b or VEGF_165_ binding to the anti-VEGF_165_b antibody. (**A**) VEGF_165_b bound with relatively high affinity and dissociation constants (2.9 × 10^4^ M^−1^ s^−1^ and 0.011 s^−1^, respectively). (**B**) Even at 180 nM,VEGF_165_ showed no binding to the antibody.

The expression of VEGF_*xxx*_b was examined by immunohistochemistry using a mouse monoclonal IgG_1_ antibody raised to the terminal 9 amino acids of the VEGF_165_b sequence. It is affinity purified against the antigen from conditioned media of hybridoma cells, and is commercially available from R&D systems (Minneapolis, MN, USA) (cat. no. MAB3045). It has previously been shown to have specificity for the VEGF_165_b isoform over the VEGF_165_ isoforms, and does not detect VEGF_165_, or VEGF_121_, recombinant protein (Woolard *et al*, 2004; Perrin *et al*, 2005). It does detect proteins consistent with VEGF_*xxx*_b isoforms such as VEGF_121_b, VEGF_165_b, VEGF_145_b and VEGF_189_b in human tissues. Surface plasmon resonance analysis showed that the affinity of the antibody for VEGF_165_b was 3.7 × 10^−7^ M (Figure 2A), whereas it had no detectable binding to VEGF_165_ (Figure 2B). Sections were prepared as above, and antigen retrieval was performed as above, but in 0.02 M sodium citrate buffer, pH 6.0. Sections were immersed in 3% hydrogen peroxide for 5 min to block endogenous peroxidase. Then they were incubated with 1.5% NHS for 30 min in a humid chamber at room temperature to block nonspecific binding. After washing, each slide was subject to 60 *μ*l of 4 *μ*g ml^−1^ MAB3045 anti-VEGF_165_b monoclonal antibody diluted in PBS/T–1% BSA overnight at 4°C. The control received a matched concentration of normal mouse immunoglobulin diluted in PBS/T–1% BSA. The blocking step with NHS was repeated before the addition of the biotinylated horse anti-mouse secondary antibody (Vector BA-2000, 25 *μ*g ml^−1^ in NHS; 1 : 66 in PBS) for 30 min. Then avidin-biotinylated enzyme complex was prepared and incubated with sections for 30 min at room temperature. This was followed by the addition of the visualising DAB substrate and counterstaining in haematoxylin as above. Sections were dehydrated in graded alcohols (70, 90 and 100% ethanol) before final clearing in xylene and mounting with DPX and glass coverslips. Slides were independently assessed and staining intensity scored (0 to 3+) as has been previously described for VEGF staining in melanoma (Potti *et al*, 2003, 2004). Light microscopy examination was performed by three different assessors, blinded to the metastatic outcome of these tumours or identity of the primary antibody. They were asked to grade the intensity of staining (0–3) in the horizontal growth phase (HGP), vertical growth phase (VGP) and peritumoral areas of each section. The metastatic and nonmetastatic tumours were matched for thickness, patient age and ulceration, and the staining intensity compared.

To determine the relative intensity of the VEGF isoforms in metastatic melanoma, the staining intensity in the melanoma (either VGP or HGP, on a scale of 0–3) was normalised to that in the peritumoral (histologically normal) skin for each stain. The ratio of the two scores was then calculated (pan-VEGF/VEGF_*xxx*_b).

## RESULTS

**Figure 3:**
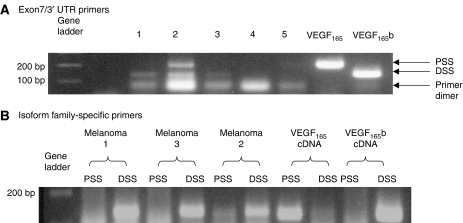
Distal and proximal splice variants of VEGF are found in archival melanoma tissues. (**A**) Extraction of mRNA from 8- to 10-year-old archival sections of melanoma and surrounding skin followed by RT–PCR using primers in exon 7 and the 3′ UTR of the VEGF gene resulted in two products of 150 and 200 bp. These were consistent with VEGF_165_b and VEGF_165_, respectively. (**B**) Splice site-specific primers to the proximal splice site (PSS) and the distal splice site (DSS) confirmed expression of these isoforms in archival sections.

Reverse transcription–PCR of archival tissue showed that both VEGF_165_b and VEGF_165_ were expressed in melanoma sections (Figure 3). Expression varied, possibly owing to variable efficiency of RNA extraction, but VEGF_165_b was seen in most samples and VEGF_165_ in some. To confirm that these isoforms were generated from DSS selection and proximal splice site (PSS) selection, PCR was carried out using isoform-specific primers. Figure 3B shows expression of VEGF_165_b in three melanoma samples, whereas VEGF_165_ was clearly seen in only one sample (melanoma number 2). It was therefore clear that VEGF_165_b was expressed in sections of human melanoma. However, these sections contained three different types of tissue, and mRNA was extracted from all three – normal skin, HGP melanoma and VGP melanoma. To localise the expression of the isoforms, we performed immunohistochemistry using a VEGF_*xxx*_b-specific antibody.

**Figure 4:**
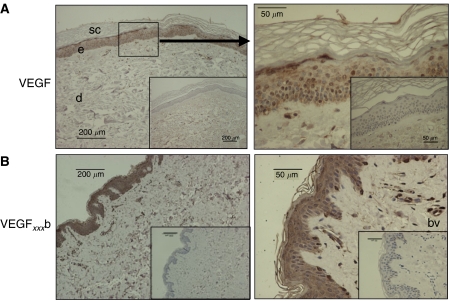
Expression of VEGF in normal skin. (**A**) Expression of VEGF determined by staining with antibodies that detect all isoforms (pro- and anti-angiogenic) of VEGF (pan-VEGF antibody). Staining is seen in epidermis (e) and weak diffuse expression in dermis (d) and around blood vessels (bv). Inset is negative control. (**B**) Expression of VEGF_*xxx*_b in skin. Expression was seen in epidermis, with very weak staining in dermis. Blood vessels were positive for VEGF_*xxx*_b. Scale bars are already given in the figure.

Pan-VEGF staining of normal skin revealed VEGF expression throughout the epidermis and only weak staining in the dermis (Figure 4A). At higher magnification (Figure 4A; right-hand picture), staining was also seen around blood vessels. Staining with a VEGF_*xxx*_b-specific antibody revealed that in normal skin VEGF_*xxx*_b was expressed in the same places as the pan-VEGF stain – namely strong expression in the epidermis, with weaker staining in the dermis and blood vessels (Figure 4B).

**Figure 5:**
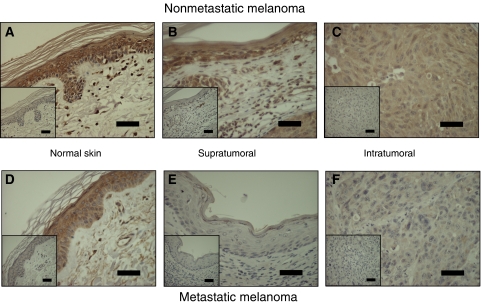
Expression of VEGF_*xxx*_b in metastatic and nonmetastatic melanoma and surrounding skin. Expression of VEGF_*xxx*_b was lower in horizontal (supratumoral) and vertical (intratumoral) growth phase metastatic melanoma than in nonmetastatic melanoma or normal skin. (**A**–**C**) Nonmetastatic, (**D**–**F**) metastatic melanoma. (**A** and **D**) Histologically normal skin >1 mm horizontally from tumour, (**B** and **E**) horizontal growth phase (supratumoral), (**C** and **F**) vertical growth phase (intratumoral). Scale bars 50 *μ*m.

Figure 5 compares expression in and around a melanoma that did not metastasise with the one that metastasised. In nonmetastatic melanoma (Figure 5A–C), expression of VEGF_*xxx*_b in the peritumoral epidermis (greater than 1 mm from the tumour margin; Figure 5A) and in the HGP (supratumoral; Figure 5B) appeared to be the same as in normal skin. In the VGP (intratumoral) of nonmetastatic melanoma, VEGF_*xxx*_b appeared to be diffusely and widely expressed, similar to normal dermis (Figure 5C). In metastatic melanoma, however (Figure 5D–F), although VEGF_*xxx*_b expression appeared similar to normal dermis in the peritumoral epidermis (Figure 5D), it was not expressed in either the HGP (Figure 5E) or the VGP (Figure 5F).

**Figure 6:**
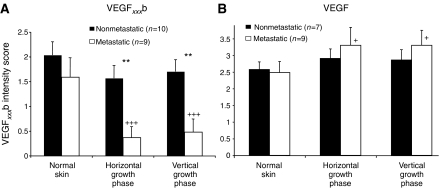
Staining of VEGF_*xxx*_b but not that of VEGF is reduced in metastatic but not in nonmetastatic melanomas. (**A**) Mean±s.e.m. score from three independent observers blinded to metastatic status of staining intensity of images of melanoma samples. (**B**) Pan-VEGF was not altered in nonmetastatic melanoma, but, in contrast to VEGF_*xxx*_b, appeared to increase in metastatic melanoma from normal through horizontal growth phase (HGP) to vertical growth phase (VGP). ^*^=Nonmetastatic *vs* metastatic, VEGF_*xxx*_b, *P*<0.0001 one-way analysis of variance (ANOVA), ^**^=*P*<0.01 Bonferroni. ^+^=Compared with normal skin. VEGF_*xxx*_b: metastatic, *P*<0.0001 one-way ANOVA, pan-VEGF: metastatic, *P*<0.05, ANOVA.^+^=*P*<0.05 ^+++^=*P*<0.001, Dunnetts.

To determine whether objective scoring of the expression intensities was different between metastatic and nonmetastatic samples, sections were assessed by three independent observers on an integral scale of 0–3 (3 being the highest intensity of staining), blind to metastatic outcome. Figure 6A shows that there was a significant downregulation of VEGF_*xxx*_b staining intensity in metastatic compared with nonmetastatic melanoma (*P*<0.001, Kruskal–Wallis) both deep within (VGP 0.48±0.26 (metastasis) *vs* 1.7±0.24 (nonmetastasis), *P*<0.05 Dunn's) and on the surface (HGP 0.37±0.2 (metastasis) *vs* 1.57±0.25 (nonmetastasis), *P*<0.05 Dunn's) but not in peritumoral epidermis (2.0±0.27 (nonmetastasis) *vs* 1.6±0.38, *P*>0.05 Dunn's). This finding held true for both thick and thin melanoma.

**Figure 7:**
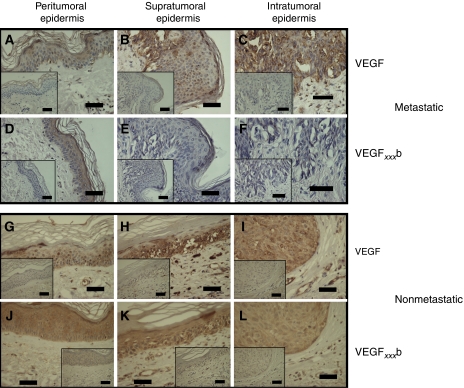
In metastatic melanomas, VEGF staining, but not VEGF_*xxx*_b staining is upregulated. Although VEGF staining was clearly seen in peritumoral epidermis, and horizontal growth phase (HGP; supra) and vertical growth phase (VGP; intra), this was in contrast to VEGF_*xxx*_b staining in serial sections. In nonmetastatic melanomas however, VEGF staining was similar to VEGF_*xxx*_b staining in all three tissue types. (**A**–**F**) Metastatic melanoma, (**G**–**L**) nonmetastatic melanoma, (**A**–**C** and **G**–**I**) VEGF staining, (**D**–**F** and **J**–**L**) VEGF_*xxx*_b staining, (**A**, **D**, **G** and **J**) normal skin, (**B**, **E**, **H** and **K**) HGP(supratumoral), (**C**, **F**, **I** and **L**) VGP (intratumoral). Scale bars=50 *μ*m.

**Figure 8:**
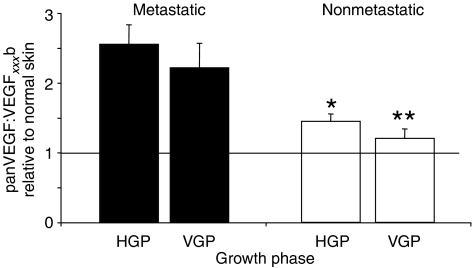
Relative levels of panVEGF to VEGF_*xxx*_b staining expression are decreased in metastatic melanoma. The normalised ratio of VEGF_*xxx*_b to VEGF staining was significantly reduced in metastatic compared with nonmetastatic melanoma (^*^=*P*<0.05, ^**^=*P*<0.01 compared with metastatic, *n*=9 metastatic, *n*=7 nonmetastatic).

To compare total VEGF expression with VEGF_*xxx*_b expression, serial sections of 16 of the 19 melanomas matched for thickness, ulceration and patient age were stained with the pan-VEGF antibody. In nonmetastatic melanoma, expression of VEGF in the peritumoral epidermis (Figure 7A), HGP (Figure 7B) and VGP (Figure 7C) appeared to be similar to that in normal epidermis or dermis as appropriate. In metastatic melanoma, however, there was increased intensity of staining from peritumoral epidermis (Figure 7D) to HGP (Figure 7E) and then to VGP (Figure 7F). This was in complete contrast to VEGF_*xxx*_b staining. Blind scoring of these melanomas showed that pan-VEGF expression was slightly but significantly increased in VGP metastatic melanomas (*P*<0.05; ANOVA; Figure 6B) but not in the nonmetastatic melanomas. To determine whether the ratio of the two stains (pan-VEGF:VEGF_*xxx*_b) was significantly different, the scores for each tumour relative to normal skin were normalised to the VEGF_*xxx*_b score relative to normal skin for that tumour. Figure 8 shows that there was a significantly higher ratio of pan-VEGF to VEGF_*xxx*_b in metastatic (*n*=9) than in nonmetastatic melanomas (*n*=7).

## DISCUSSION

The ability to accurately determine which patients will go on to develop metastasis is a key goal in current melanoma therapy. Patients are currently followed up every 3 months for 3 years, even after removal of thin tumours, as a significant proportion (15%) of these will go on to develop metastasis. Patients with thicker melanoma (> 1mm) will be followed for a further 2 years at 6 monthly intervals (Roberts *et al*, 2002). Recent studies have identified lymphatic density of the primary tumour upon excision as a potential predictor of metastasis (Dadras *et al*, 2003; Shields *et al*, 2004). This increased lymphatic density may be brought about by growth of new lymphatics towards tumours expressing VEGF-C or VEGF-D (Skobe *et al*, 2001), by stochastic variation of melanomas or metastatic chemotaxis towards lymphatic endothelial cells (Shields *et al*, 2007). The identification of increased lymphatic density has been combined with thickness and lymphatic invasion to generate a prognostic index (Shields' index) that predicts the likelihood of metastasis, at least in a relatively small number of patients (Shields *et al*, 2004). Other indicators, such as lymphatic hot spots, have also been used (Dadras *et al*, 2003, 2005). Both these mechanisms require the staining and counting of lymphatic surrounding tumours, and are labour intensive, and in the case of hot spots subjective. The identification of molecules shown to be downregulated in melanoma would make identification of potentially metastatic tumours easier.

The antibody used here is an affinity-purified monoclonal antibody, and has been shown using Western blotting (Woolard *et al*, 2004; Perrin *et al*, 2005), immunohistochemistry (Woolard *et al*, 2004) and ELISA (Woolard *et al*, 2004; Bates *et al*, 2006) to detect only the VEGF_*xxx*_b isoforms of VEGF. Samples extracted from various human tissues, when probed with this antibody, reveal multiple bands of the same size as that detected using a pan-VEGF antibody – although at different relative intensities. It has so far been shown to detect VEGF_121_b, VEGF_165_b, VEGF_189_b, VEGF_183_b and VEGF_145_b but none of the VEGF_*xxx*_ isoforms, even when present at 10 000-fold greater concentrations (Perrin *et al*, 2005).

The downregulation of the VEGF_*xxx*_b isoforms identified here, if confirmed in a larger study, would greatly simplify the procedure for identifying patients at risk. Furthermore, it could be combined with other indices, such as the Shields' index, to further refine metastatic prediction (Shields *et al*, 2004). However, the mechanisms underlying downregulation of VEGF_*xxx*_b isoforms have not been identified here. Possible explanations of this finding include the following: (1) VEGF_*xxx*_b expression inhibits tumour metastasis directly by interfering with tumour cell migration or tumour cell adhesion via a currently unknown mechanism; (2) inhibition of angiogenesis by VEGF_165_b expression (Woolard *et al*, 2004) is responsible for preventing metastasis by limiting tumour size independently of thickness; (3) VEGF_*xxx*_b expression inhibits the main route of metastatic spread via the lymphatics by inhibition of lymphangiogenesis; (4) increased vascular permeability induced by VEGF_*xxx*_ increases the likelihood of metastasis; or (5) a combination of any or all of the above factors.

VEGF_*xxx*_b has been shown to be anti-angiogenic in physiological models, and inhibits melanoma xenograft growth *in vivo* (Woolard *et al*, 2004; Konopatskaya *et al*, 2006), but results in a very transient increase in vascular permeability to water (Glass *et al*, 2006), whereas VEGF_165_ is angiogenic and results in a chronic and sustained increase in water permeability (Senger *et al*, 1983, 1990; Bates and Curry, 1996), leading to oedema in many tumours. Upregulation of VEGF_165_ with respect to VEGF_165_b will therefore result in angiogenic, leaky tumours, and it is likely that this would provide a more facilitative environment for metastasis for a number of reasons. These include a more hydrated tissue, which would be easier for cell and molecules to move through (Criscuoli *et al*, 2005). This would result in tumour cells having a greater likelihood of detecting lymphatic-secreted chemokines to identify the lymphatics (Podgrabinska *et al*, 2002), and being able to secrete heparin-binding growth factors a further distance to stimulate lymphatic growth into the tumours. Recent studies have also shown that lymphatic cells can migrate along patterns of interstitial fluid flows (Boardman and Swartz, 2003), and presumably this would be enhanced in more permeable tumours. Thus, the expression characteristics of these tumours indicate that upregulation of pro-angiogenic, pro-permeability VEGF_165_ and its sister isoforms is associated with metastasis in melanoma. The ratio of pan-VEGF to VEGF_*xxx*_b staining shown in Figure 8 is qualitatively similar to an angiogenic ratio that can be calculated from quantitative assessment of VEGF isoforms by ELISA. This ratio reflects the angiogenic balance of VEGF isoforms and can be considered an angiogenic ratio. For quantitative studies, a value of 1 would indicate that all the VEGF is VEGF_*xxx*_b, whereas values above 1 indicate the presence of non-VEGF isoforms, and values greater than 2 indicate a pro-angiogenic state. For semiquantitative assessments such as those described here, the degree of the angiogenic switch cannot be assessed, but it is clear that the metastatic tumours have a more angiogenic balance of isoforms than the nonmetastatic tumours.

These findings also show that the metastatic process appears to be associated with a splicing switch. Recently, the role of splicing in cancer has received a good deal of attention (Venables, 2004), but it is clear that alternative splicing events also play a role in the metastatic process (Venables, 2006; Tsuji *et al*, 2006). The regulation of VEGF splicing may therefore also be part of a metastatic splicing phenotype that is regulated by specific splice factors, such as SF/ASF2, described recently for the macrophage-stimulating promoter receptor tyrosine kinase Ron (Ghigna *et al*, 2005), and for which we also have evidence that it is involved in the regulation of VEGF_*xxx*_:VEGF_*xxx*_b splicing (Woolard *et al*, 2006).

In summary, we have shown that VEGF_*xxx*_b proteins are downregulated in metastatic but not in nonmetastatic malignant melanomas, but the mechanism underlying this is not known. This could result in a greater accuracy of prognosis for metastatic melanoma.

## Figures and Tables

**Figure 1:**
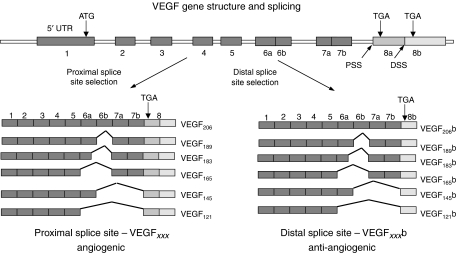
Alternative splicing of the VEGF gene in the terminal exon results in two families of isoforms – the angiogenic VEGF_*xxx*_ and the anti-angiogenic VEGF_*xxx*_b isoforms.
